# Development of a simple trap using widely available materials for collecting immature Sabethines (Diptera: Culicidae) and first records of *Sabethes batesi* and *Wyeomyia nigritubus* in Sergipe, Brazil

**DOI:** 10.1590/0037-8682-0107-2025

**Published:** 2025-12-15

**Authors:** David Campos Andrade, João Victor Santos França, Rafael Oliveira Christe, Mauro Toledo Marrelli, Roseli La Corte

**Affiliations:** 1 Universidade Federal de Sergipe, Departamento de Morfologia, São Cristóvão, SE, Brasil.; 2 Universidade de São Paulo, Faculdade de Saúde Pública, São Paulo, SP, Brasil.

**Keywords:** Culicidae, Sabethini, Biogeography, Entomological trap, Atlantic forest, Yellow fever

## Abstract

**Background:**

The collection of immature Sabethines from natural habitats is challenging. Objective: To develop a low-cost and effective trap using widely available materials.

**Methods:**

A perforated coconut endocarp filled with water was installed in the Atlantic Forest of Sergipe.

**Results:**

*Sabethes albiprivus*, *Sa. batesi*, *Toxorhynchites theobaldi*, and *Wyeomyia nigritubus* were identified after collection and rearing.

**Conclusion:**

The traps proved effective in collecting immature Sabethines and contributed to expanding the geographic range of *Sa. batesi* and *Wyeomyia nigritubus*. This method offers a new alternative for collecting sylvatic yellow fever vectors.

Sabethines constitute a diverse group of primarily sylvatic and diurnal mosquitoes that utilize various phytotelmic habitats to lay eggs. Sabethini species became better known in Brazil through research conducted by the Yellow Fever Prophylaxis Service during the first half of the 20th century, which addressed the first disease to be subject to mandatory notification in the country^1^ ^,^ ^2^. However, since sampling efforts were largely associated with disease outbreaks, the distribution and role of some species in maintaining the yellow fever cycle are still not well understood, particularly in the Northeast Brazil^3^.

The choice of habitat, egg-laying behavior, or host preference for hematophagy may be exclusive to many mosquito species. Plant axils, fallen leaves, flowers, fruit husks, tree holes, and internodes of bamboo are known sites for oviposition and larval development^1^ ^,^ ^4^. In response to these preferences, various traps, both artificial, such as the ovitrap, and natural, such as bamboo internodes (stems) filled with water, have been developed for mosquito collection^5^.

More recently, the fruits of the sapucaia tree (*Lecythis pisonis* Cambess) have been successfully used for the collection of sylvatic mosquitoes, particularly species of the genus *Sabethes*, which were subsequently maintained in laboratory conditions for vector competence studies^6^. The fruit is globose and operculate with a thick rind. However, the distribution of *L. pisonis* is not uniform throughout Brazil; for example, this species is not found in the state of Sergipe, thereby making wide adoption of this technique difficult^7^.

Thus, based on the natural collection tools mentioned above (bamboo and sapucaia), another simple, low-cost alternative was developed using the fruit of the coconut tree (*Cocos nucifera* Linn.), a widely available material. The husk of this fruit has been documented as a breeding site^1^. Coconut trees are widely distributed in Brazil, and although of Asian origin, the favorable climate facilitated the introduction of this species during the colonization process, and it is now used in various economic activities^8^.

The new technique involves the use of the decomposed fruit, leaving only the mature endocarp, commonly known as the “coconut ball.” These were collected from areas surrounding coconut plantations (Figure 1A). The “coconut ball” is the structure that remains after the natural decomposition of the epicarp, mesocarp, and endosperm (pulp) or after the consumption of these parts by other organisms (Figure 1B). The endocarp of *C. nucifera* is solid and resistant because it is composed mainly of cellulose, hemicellulose, and lignin and serves as a natural reservoir for water stored by the fruit^9^.

The adaptation of the “coconut ball” proved to be simpler than that of the sapucaia fruit, as it already has three germination pores. Two of these natural pores were used to thread a string and hang the trap from tree branches, whereas the third one was used to fill the trap with water and empty it in the field (Figure 1C). The fruit does not require waterproofing due to the hardness of its endocarp^5^ ^,^ ^6^. A series of 5-8 holes, approximately 2-4 mm in diameter, were made along the circumference of the coconut ball at its midsection to mimic the pattern of perforations left by female beetles (Coleoptera: *Curculionidae*) in bamboo, as described by Lima Bersot et al. (2023)^10^ (Figure 1B). In addition, this row of holes served as a reference point for the water level inside the trap, as the excess water overflowed through these openings during filling.

**FIGURE 1:**
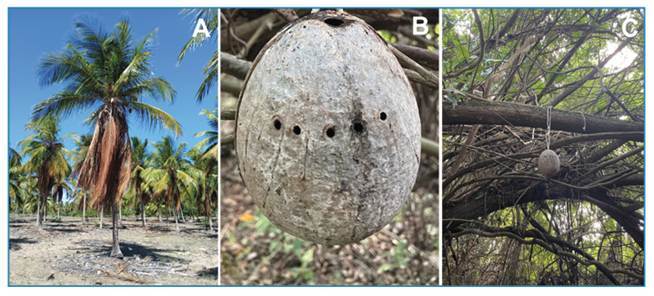
Trap made with coconut endocarp. **A:** *Cocos nucifera* plantation; **B:** Fruit endocarp adapted for collecting immature mosquitoes; **C:** Trap installed in the field.

The traps were installed for testing at two locations in a remnant of the Atlantic Forest in the municipality of Santa Luzia do Itanhy in Sergipe, Brazil (Location 1:11°23'36.8"S, 37°25'31.2"W; Location 2: 11°22'45.9"S, 37°25'22.3"W). Initially, two traps were installed, and after a pilot test of thirty days, two more traps were added at each location, making a total of six. They remained in the field from July to September 2024 and were inspected every two weeks, for a total of six visits. The water was changed through the germination pores, which were not sealed during field testing. Within the first two weeks after installation, the traps were already colonized. All aquatic contents were taken to the Laboratory of Entomology and Tropical Parasitology (LEPaT) at the Federal University of Sergipe, even if larvae were not present, as some samples contained Culicidae eggs that hatched in the laboratory a few days after collection.

A total of 67 immature larvae or eggs were collected, raised to adulthood in the laboratory, and identified as: *Sabethes albiprivus* Theobald, 1903 (22♀;21♂), *Sa. batesi* Lane & Cerqueira, 1942 (6♀; 4♂); *Toxorhynchites theobaldi* Dyar & Knab, 1906 (1♀;1 larva 4^th^ ínstar), and *Wyeomyia nigritubus* Galindo, Carpenter & Trapido, 1951 (8♀;4♂), according to the keys^1^ ^,^ ^3^ ^,^ ^4^ using male genitalia, larval exuvia, and pupae when necessary. Specimens of each Sabethine species have been deposited in the LEPaT entomological collection: *Wy. nigritubus* (00010♀, 00011♂); *Sa. batesi* (00012♀, 00013♂), and *Sa. albiprivus* (00014♀, 00015♂). *Sabethes albiprivus* has already been recorded in Sergipe and has epidemiological relevance in the transmission of yellow fever^3^. 

*Wyeomyia nigritubus* was first described in bamboo samples in Panama in 1951^11^. The male genitalia of this species were similar to those of *Wy. caracula* (Dyar & Nuñez Tovar, 1927), which was described in adults collected from bromeliads in Venezuela^4^. In 1970, during a revision of the species from Jamaica, Belkin et al. (1970)^12^ noted that the redescription of *Wy. caracula* made by Hill and Hill (1945)^13^ based on bamboo specimens from Jamaica, including both immature and male genitalia, actually referred to *Wy. nigritubus*. Lane (1953)^4^ used redescriptions, including reproductions of the original drawings, to develop his key^4^ ^,^ ^13^. Therefore, according to Lane’s key for male genitalia, *Wy. caracula*, a species associated with bromeliads, could be mistakenly identified when the specimen is actually *Wy. nigritubus*, a species found in tree holes and bamboo^1^ ^,^ ^11^ ^-^ ^13^. *Wy. caracula* was recorded once in the northern region of Brazil in 2011^14^, and this is the first record of *Wy. nigritubus* in Brazil. In our survey, both immature forms (larvae with well-sclerotized air tubes) and male genitalia of *Wy. nigritubus* are in good agreement with the description, but the adult forms differ, as they lack white markings on the middle leg, which are present only on the fourth and fifth tarsi of the hind leg in females and partially extend to the third tarsus in males (Figure 2).

**FIGURE 2:**
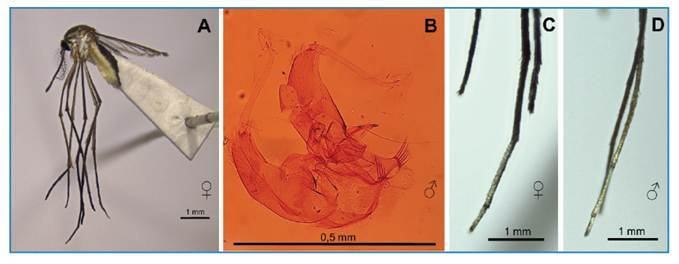
*Wyeomyia nigritubus*: **A:** Female adult; **B:** Male genitalia; **C:** White markings on the tarsae of the female's hind leg; **D:** White markings on the tarsae of the male's hind leg.

The species *Sabethes batesi* (Figure 3A,B,C ) was described by Lane & Cerqueira in 1942^1^ based only on males and named in honor of the British naturalist Henry Walter Bates, who contributed to the field of biology with respect to camouflage and mimicry. However, it is difficult to differentiate adult females from *Sa. cyaneus* (Fabricius, 1805)^3^. It is possible that the females of *Sa. batesi* were not described^1^ due to their strong similarity to related species, which may have also affected the recognition of their distribution and contributed to their absence in studies on natural arbovirus infection^3^. *Sabethes cyaneus* occurs in several countries in the American continent and has been reported to have epidemiological relevance, whereas *Sa. batesi* has only been recorded in five Brazilian states, including the results of the current study, which extends its occurrence to the Northeast Brazil^1^ ^,^ ^3^.

Initially, it was suggested that the distinction between the two adult species could be made based on the length of the upper mesepimeral bristles; however, there was disagreement among taxonomists regarding this length. A recently updated key for the genus *Sabethes* established that the lengths of these bristles in the two species were similar^1^ ^,^ ^3^ ^,^ ^4^. In their updated key, Neves et al. (2024)^3^ suggested that the species can be distinguished by differentiation in the shape of the lateral scales that divide the colors in abdominal segments VI and VII, being in a straight line for *Sa. batesi* or rounded incisions for *Sa. cyaneus*.

Although Neves et al. (2024)^3^ did not indicate the existence of upper proepisternal bristles in any of the species of the *Sabethes* subgenus, the specimens recorded in Sergipe had two of them, albeit inconspicuous (Figure 3D ). This observation, made by Harbach (1992)^15^, may help distinguish between species, although bristles may be absent in some cases^15^. However, male genitalia allow for a clear distinction between species^4^.

**FIGURE 3:**
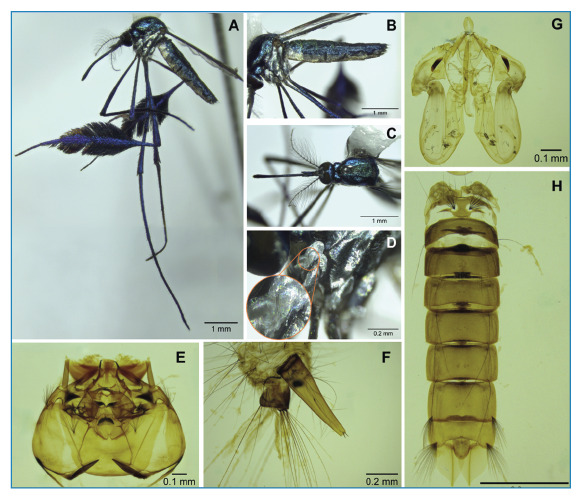
*Sabethes batesi*: **A,B,C:** Adult specimen from different perspectives; **D:** Proepisternal bristles; **E:** Larval head; **F:** Larval siphon; **G:** Pupal cephalothorax; **H:** Pupal abdomen.

The collection of *Sabethes batesi* in the adult form is rare^3^. The distances between the states where the specimens of *Sa. batesi* were previously collected, including the North, Southeast, and South regions of the country, and now also the Northeast region, suggest a broad distribution of this species in Brazil. The low number of adult specimens collected in surveys may be due to possible misidentification, particularly of females, as previously mentioned, or may be related to the species' bionomy, either due to its preference for hematophagy within specific vegetational strata or a possible low attraction to human hosts. For oviposition, *Sa. batesi* have a preference for traps that have narrow hole (for example, bamboo and sapucaya)^3^. Due to the lack of information in the literature on the aquatic phases of *Sa. batesi,* we included photographs of the larval and pupal exuviae (Figure 3E,F,G,H ) to contribute to future research^1^ ^,^ ^4^.

The coconut ball trap proved to be a simple, effective, and low-cost method for collecting immature *Sabethes*, making it useful for the surveillance of sylvatic vectors of the yellow fever virus and taxonomic studies of the group. During this period of only a few months in the field, this group demonstrated selectivity and collected a considerable number of larvae (67). In a study by Dias et al. (2024)^16^, who tested several traps for yellow fever vectors over more than two and a half years, with four traps installed monthly, traps in natural containers, such as sapucaia and bamboo internodes, produced 237 and 222 larvae, respectively, over the entire period. These results confirm that the coconut ball trap is a useful complementary tool for sampling sylvatic mosquito immatures.

Depending on the objective of the study and trap time in the field, it may be advisable to seal the upper hole of the germinal bud and individualize the specimens for their associated rearing. Adjustments, such as different shapes and sizes of the holes, vertical distribution of the traps, and degree of fruit maturation, could also be evaluated. Enlarging the holes may result in a different species composition, as observed with tree hollows, similar to what was detected by Vieira et al. (2020)^6^ in sapucaya.

In addition to the methodological contribution to entomological surveillance^5^, collection of immature sylvatic culicids enables accurate taxonomic identification^1^ ^,^ ^4^. This approach yielded findings with public health implications, including the first record of *Sabethes batesi* in the Northeast Brazil, expanding our knowledge of the biogeographic distribution of potential arbovirus vectors^3^. Similarly, confirmation of *Sa. albiprivus*, a species with recognized relevance for yellow fever transmission^3^ ^,^ ^6^, underscores the vulnerability of both humans and non-human primates in the event of a viral introduction into this remnant of the Atlantic Forest. Collectively, these findings emphasize the value of disseminating innovative and low-cost monitoring tools to expand vector surveillance in neglected areas.

## Data Availability

All data are contained in the main text.
